# Identification and Tumour-Binding Properties of a Peptide with High Affinity to the Disialoganglioside GD2

**DOI:** 10.1371/journal.pone.0163648

**Published:** 2016-10-07

**Authors:** Jan Müller, Robin Reichel, Sebastian Vogt, Stefan P. Müller, Wolfgang Sauerwein, Wolfgang Brandau, Angelika Eggert, Alexander Schramm

**Affiliations:** 1 Pediatric Oncology and Hematology, University Children’s Hospital Essen, University of Duisburg-Essen, Essen, Germany; 2 Nuclear Medicine, University Hospital Essen, University of Duisburg-Essen, Essen, Germany; 3 Radiation Oncology, University Hospital Essen, University of Duisburg-Essen, Essen, Germany; 4 Pediatric Oncology and Hematology, Charité Universitätsmedizin, Berlin, Germany; Consejo Superior de Investigaciones Cientificas, SPAIN

## Abstract

Neuroectodermal tumours are characterized by aberrant processing of disialogangliosides concomitant with high expression of GD2 or GD3 on cell surfaces. Antibodies targeting GD2 are already in clinical use for therapy of neuroblastoma, a solid tumour of early childhood. Here, we set out to identify peptides with high affinity to human disialoganglioside GD2. To this end, we performed a combined in vivo and in vitro screen using a recombinant phage displayed peptide library. We isolated a phage displaying the peptide sequence WHWRLPS that specifically binds to the human disialoganglioside GD2. Binding specificity was confirmed by mutational scanning and by comparative analyses using structurally related disialogangliosides. In vivo, significant enrichment of phage binding to xenografts of human neuroblastoma cells in mice was observed. Tumour-specific phage accumulation could be blocked by intravenous coinjection of the corresponding peptide. Comparative pharmacokinetic analyses revealed higher specific accumulation of ^68^Ga-labelled GD2-binding peptide compared to ^111^In-labelled peptide in xenografts of human neuroblastoma. In contrast to ^124^I-MIBG, which is currently evaluated as a neuroblastoma marker in PET/CT, ^68^Ga-labelled GD2-specific peptide spared the thyroid but was enriched in the kidneys, which could be partially blocked by infusion of amino acids.In summary, we here report on a novel tumour-homing peptide that specifically binds to the disialoganglioside GD2, accumulates in xenografts of neuroblastoma cells in mice and bears the potential for tumour detection using PET/CT. Thus, this peptide may serve as a new scaffold for diagnosing GD2-positive tumours of neuroectodermal origin.

## Introduction

Neuroblastoma is the most common extracranial solid tumour of childhood. Its accounts for more than 7% of malignancies in children younger than 15 years and approximately 15% of cancer related mortality in paediatric oncology patients. Neuroblastoma is a disease of the sympathoadrenal lineage of the neural crest, and therefore tumours develop at various sites along the sympathetic nervous system. Most primary tumours (65%) occur within the abdomen, and the majority of these arise in the adrenal medulla. Other common sites of disease include neck, chest, and pelvis [1, 2]. Few recurrent genetic events have been linked to neuroblastoma including tumour-specific amplification of the MYCN oncogene or mutations in the genes coding for anaplastic-lymphoma kinase (ALK) and the alpha thalassemia/mental retardation syndrome X-linked (ATRX) gene [3, 4]. Recently, activation of telomerase by genomic rearrangements has been identified as a hallmark of an aggressive subtype of neuroblastoma [5]. However, the clinical problem in neuroblastoma is the lack of therapeutic options in relapsing disease [6], which has been linked to significantly increased mutational burden and RAS pathway activation [7, 8]. Thus, novel strategies for therapy and monitoring disease status of neuroblastoma are eagerly awaited.

Interestingly, neuroblastoma tumour cells are characterized by expression of the disialoganglioside, GD2, on their surface. Generally speaking, disialogangliosides are part of a heterogeneous family of gangliosides. These glycosphingolipids consist of a ceramide lipid moiety and a linked glycan chain of varying length and structure. The disialogangliosides GD1b, GD2 and GD3 are all composed of glucose linked to ceramide and a galactose, but differ in their terminal moieties [9]. GD2 is found on cells of neuroectodermal origin and its physiological distribution in humans is limited to neurons and peripheral nerve fibres [10]. GD2 expression in tumours is not restricted to neuroblastoma, but was also found on different tumour cells and tissues, for example melanoma [11], osteosarcoma [12], uterine leiomyosarcoma [13], small cell lung cancer [14], Ewing sarcoma [15], retinoblastoma [16] and others [17]. GD2 is present on almost all cell membranes of NB tumours regardless of stage, and is abundantly expressed with an estimated 5–10 million molecules/cell [18]. It is suggested that shedding of GD2 and MYCN amplification jointly characterize the most aggressive type of neuroblastomas [19]. Antibodies directed against GD2 inhibit neuroblastoma cell attachment to extracellular matrix components like collagen, vitronectin, laminin and fibronectin [20]. Attachment to these matrix components is mediated via an Arg-Gly-Asp (RGD) motif [21]. In GD2 expressing lung cancer cells, the addition of a GD2 specific antibody caused growth reduction and apoptosis [22]. Ko and co-workers demonstrated the invasive potential of GD2 positive lung cancer cells in contrast to GD2 negative cells [23], suggesting a role for GD2 also in metastatic processes. Taken together, GD2 can contribute to cell growth, attachment to extracellular matrix components and enhance cell migration in different tumour entities.

Several murine, chimeric and humanized monoclonal antibodies (mAbs) have been developed to target GD2, ever since the prototypic mAb 3F8 was developed in 1985. Subsequently murine mAbs, 14G2a, and the chimeric human variant ch14.18 were used in clinical trials for the therapy of neuroblastoma [18]. Several clinical studies revealed that the combination of mAb ch14.18, IL-2 and GM-CSF or variant GD2 antibody fused to IL-2 (mAb hu14.18-IL2) showed increased efficiency to achieve long-term event-free survival of neuroblastoma patients [24, 25]. Administration of GD2 specific antibodies is sufficient to induce cell death in neuroblastoma cell lines in vitro [26, 27]. Moreover, GD2 is also a valuable target for imaging purpose, which was shown by radioactively labelled GD2 antibodies [28, 29]. Thus, GD2 has been recognised as a target both for imaging and treatment of neuroblastoma [30, 31].

Phage display screening has been widely used for identification of specific oligopeptides recognizing different biological structures. It has been shown that peptide libraries displayed on phages can be screened in vivo and in vitro for phages that home to a specific target. A number of peptides capable of homing to tumour vasculature or structures on the surface of tumour cells were isolated using phage display screenings [32–34]. Moreover, the therapeutic efficacy of these tumour-homing peptides coupled to cytotoxic drugs has been demonstrated in preclinical models of cancer [35]. These peptides could also be used for imaging purposes [36]. Here, we applied phage display technology to identify peptides that specifically bind to the human disialoganglioside GD2 and to characterise the tumour-homing ability and suitability of these peptides for imaging human neuroblastoma cells using xenograft models.

## Materials and Methods

### Materials

Human gangliosides GD2, GD3, GD1b and GT1b were purchased from HyTest (Turku, Finland). The M13 phage library, the M13KE vector DNA, the enzymes *Acc65*I and *Eag*I and the phage-host bacteria *Escherichia coli* ER2738 were obtained from New England Biolabs (Frankfurt/Main, Germany). Oligonucleotides were from MWG Biotech (Ebersberg, Germany). Peptide synthesis was performed by GL Biochem (Shanghai, China). Xylazine was acquired from Bayer (Leverkusen, Germany). Ketamine was from Pfizer (Karlsruhe, Germany). Dulbecco´s PBS (DPBS) were purchased from Invitrogen (Karlsruhe, Germany). Thiazolblue (MTT reagent) was from Roth (Karlsruhe, Germany). All other chemicals were purchased from Sigma-Aldrich (Taufkirchen, Germany).

### Cell Culture

Human IMR32 neuroblastoma tumour cells expressing the disialoganglioside GD2 [27] were purchased from DSMZ (Braunschweig, Germany) and maintained in DMEM (GlutaMax I, Gibco, Karlsruhe, Germany) containing 10% fetal calf serum, 100 IE/ml penicillin, 100 μg/ml streptomycin and 2,5 μg/ml Amphotericin B at 37°C in a humidified atmosphere containing 5% CO_2_.

### Animals

NMRI nude mice (6–8 weeks old) were obtained from and kept at the central animal care facility of the University Hospital Essen, Germany. All animal experiments were carried out in accordance with the guidelines of the German Animal Welfare Act. Physical condition of the animals was monitored daily and health and welfare were assessed using a score sheet defining criteria for termination. Animal experiments were approved and supervised by the North Rhine-Westphalia State Environment Agency (Landesamt für Natur, Umwelt und Verbraucherschutz NRW, record number 87–51.04.2010.A037).

### IMR32 Neuroblastoma Tumour Model

Human IMR32 tumour cells were injected subcutaneously (s.c.) in the right and left femoral region of anesthetized NMRI nude mice (1×10^7^ IMR32 cells in 50 μl DPBS and 150 μl Matrigel (BD)). For PET imaging, IMR32 cells were injected s.c. in the neck fold. Imaging and all other interventions were conducted on day 14 day after tumour cell inoculation.

### Combined *In Vivo/In Vitro* Phage Display Screening

96-well microtiter plates (Greiner, Frickenhausen, Germany) were coated with 10 μg GD2 or 10 μg GD1b per well for one hour at room temperature. Wells were then washed 3 times with DPBS/Tween20 (0.05%), blocked with BSA (1%)/DPBS/Tween20 (0.05%) for 1 hour at room temperature, washed again and subsequently filled with H_2_O. Plates were stored at 8°C until use. For in vitro selection, 2 x 10^11^ plaque forming units (pfu) of the unselected M13 Ph.D.-7 phage display peptide library or the phage pool of the subsequent selection rounds was incubated with DPBS/Tween20 (0.1%, 0.2%, 0,3% or 0,4%) in a GD1b coated well for 1 hour at room temperature on a shaker. The phage solution was then transferred to a well containing GD2 and incubated for 1 hour at room temperature. After washing 10 times with DPBS/Tween20 (0.1%, 0.2%, 0,3% or 0,4%), bound phages were eluted by addition of *E*. *coli* ER2738 (OD_600nm_ 0.4–0.5) for 30 minutes at 37°C. Phage amplification in 20 ml suspension containing *E*. *coli* ER2738 (hereafter referred to as phage pool) and titer determination were performed according to the manufacturer’s instructions with the modification that X-Gal was replaced by S-Gal and ferric ammonium citrate (both obtained from Sigma, Taufkirchen, Germany). For *in vivo* selection, 2 x 10^11^ pfu of the phage pool of the 3rd selection round were injected into the tail vein of a mouse with a xenograft tumour induced by inoculation of IMR32 cells (2 x 10^7^). Anaesthetised mice were perfused with DPBS 10 min after phage application. Tumours were explanted, homogenized in LB-medium and the phages were eluted with 900 μl of an *E*. *coli* ER2738 suspension at 37°C for 30 minutes in an incubator. Of this suspension, 10 μl were used for the determination of the phage titer. The residual volume was used for phage amplification in 20 ml *E*. *coli* ER2738 suspension according to the manufacturer’s instructions.

### Phage Binding Assay

Wells were coated with GD3, GD2, GD1b, GT1b or BSA (10 μg/ well each). For competition experiments, coated wells were pre-incubated with serial dilutions ranging from 10 μg to 100 pg of an 11-mer GD2-specific-peptide in DPBS for one hour at room temperature on a shaker and the supernatant was removed. In total, 1 × 10^11^ pfu of the amplified phages were incubated with DPBS/Tween20 (0.3%) for one hour at room temperature on a shaker. After washing with 10 volumes of DPBS/Tween20 (0.3%), bound phages were eluted by adding *E*. *coli* ER2738 and phage titer determination was performed as described above. Additionally, an in vivo competition assay was performed in tumour-bearing mice. Therefore, 200 μg of the GD2-specific peptide and 1×10^11^ pfu of the phage encoding the same peptide were co-injected intravenously in three IMR32 tumour bearing mice per experiment. Tumours were subsequently harvested and the phage titers were determined as described above.

### Molecular Cloning of Phage Binding Sequence Motifs and Variants Thereof

Modification of the phage binding motif was achieved by standard cloning procedures. The corresponding oligonucleotides were annealed (cf. Table 1) and separately ligated into *Acc65*I / *Eag*I cleaved M13KE-phage DNA. Ligated DNA was transformed into *E*. *coli* ER2738 using the TransformAid bacterial transformation kit (Fermentas, St. Leon-Rot, Germany) according to the manufacturer’s instructions. Positive clones were identified using LB/S-Gal/IPTG-plates. Following amplification, phage DNA was extracted using the E.Z.N.A. plasmid miniprep kit I (peqLab, Erlangen, Germany) and inserts were verified by DNA sequencing.

**Table 1:** DNA sequences of oligonucleotides used in this study.

Oligonucleotide	Oligonucleotide sequence
mut1_for	5´-GTA CCT TTC TAT TCT CAC TCT GGT CAT TGG CGT CTT CCT TCT GGT GGA GGT TC-3´
mut1_rev	5´- GGC CGA ACC TCC ACC AGA AGG AAG ACG CCA ATG ACC AGA GTG AGA ATA GAA AG-3´
mut2_for	5´-GTA CCT TTC TAT TCT CAC TCT TGG CAT GGT CGT CTT CCT TCT GGT GGA GGT TC-3´
mut2_rev	5´- GGC CGA ACC TCC ACC AGA AGG AAG ACG ACC ATG CCA AGA GTG AGA ATA GAA AG-3´
mut3_for	5´-GTA CCT TTC TAT TCT CAC TCT TGG CAT TGG CGT CTT GGT TCT GGT GGA GGT TC-3´
mut3_rev	5´- GGC CGA ACC TCC ACC AGA ACC AAG ACG CCA ATG CCA AGA GTG AGA ATA GAA AG-3´
mut4_for	5´-GTA CCT TTC TAT TCT CAC TCT GGT CAT GGT CGT CTT CCT TCT GGT GGA GGT TC-3´
mut4_rev	5´- GGC CGA ACC TCC ACC AGA AGG AAG ACG ACC ATG ACC AGA GTG AGA ATA GAA AG-3´
mut5_for	5´-GTA CCT TTC TAT TCT CAC TCT GGT CAT GGT CGT CTT GGT TCT GGT GGA GGT TC-3´
mut5_rev	5´- GGC CGA ACC TCC ACC AGA ACC AAG ACG ACC ATG ACC AGA GTG AGA ATA GAA AG-3´
mut_for	5´-GCT CCT TTT GGA GCC TTT TT-3´
mut_rev	5´-ATT CCA CAG ACA GCC CTC AT-3´
-96gIII	5´-CCC TCA TAG TTA GCG TAA CG-3´

### Distribution of Phage *In Vivo*

Of the GD2-specific phage or a control phage (GHGRLPS), 1×10^11^ pfu were injected intravenously in three IMR32 tumour bearing mice per phage after an i.p. application of the anaesthetics xylazine (16 mg/kg) and ketamine (100 mg/kg). After 10 minutes, the mice were perfused with DPBS and brain, muscle, stomach, kidneys, heart, lung, spleen, liver and tumour were explanted and weighed. Tissues were homogenized in LB-medium using an Ultra-Turrax T25 homogenizer (IKA Labortechnik, Staufen, Germany). Phages were eluted by the addition of *E*.*coli* ER2738 bacteria and the phage titers were determined as described above.

### Cell Proliferation Assay

Proliferation of the human neuroblastoma cell line IMR32 upon peptide addition was evaluated using MTT assays as described [37, 38]. Peptides were used at a concentration of 1 μg and 10 μg per well in a total volume of 200 μl per well. For each time point and each peptide concentration, experiments and controls were performed in quintuplicate.

### Radiolabeling of DOTA Peptides

^111^In was solved in HCl and a pH 3 was adjusted. DOTA peptides (100 μg) were diluted in the same volume of sodium acetate buffer (pH 5.0). Next, a 1:1 mixture (v/v) of the ^111^In /HCl solution and the peptide solution was heated to 80°C for 10 minutes before applying it to a C18 reverse-phase cartridge (Strata^™^-X 33 μm Polymeric Reversed Phase, Phenomenex, Aschaffenburg, Germany). After washing twice with 5 ml water, elution of the ^111^In–labelled peptide was achieved using ethanol abs. and volume was adjusted to 100 μl using isotonic NaCl solution.

### Distribution of ^111^In -Labelled and ^68^Ga -labelled DOTA Peptides *In Vivo*

^111^In—labelled DOTA peptides (200–700 kBq) or ^68^Ga—labelled DOTA peptides (80–400 kBq) were injected intravenously into mice bearing IMR32-induced (2 x 10^7^ cells s.c.) xenografts. Organs and blood of mice were collected between 5 min and 24 h after injection and their radioactive content was quantified using a gamma counter (Automatic Gamma Counter 1480 Wizard TM 3”). The injected dose was calculated by weighing the syringe with and without the tracer. Activity concentrations were corrected for radioactive decay. To evaluate the effects of kidney protection, a mixture containing Gelofusine (80μg/ g bodyweight)/lysine (400μg/ g weight)/arginine (400μg/ g weight) was injected i.v. 5 minutes before application of the tracer. Here, organs and blood were collected 1 h after injection. To compare ^68^Ga -labelled peptides with the clinically established tracer, one group of tumour bearing mice received ^124^I -MIBG (100–200 kBq) by intravenous injection.

### PET/MRT Imaging of ^68^Ga -Labelled DOTA Peptides

For PET imaging, ~ 7 MBq of ^68^Ga -labelled DOTA peptide were injected intravenously into tumour bearing mice using kidney protection as described above. After 1 h, mice were anesthetized by i.p. application of Fentanyl (1 μg/kg), Midazolam (0.1 mg/kg), Medetomidine (10 μg/kg) and imaged using a PET/MRT scanner (Siemens Biograph mMR). PET images were reconstructed by OSEM (ordered subset expectation maximization) reconstruction with 3 iterations, 21 subsets using a zoom of 4 in a 344 matrix, and a Gaussian 2 mm FWHM post reconstruction filter.

### Statistical Analysis

Results are reported as arithmetic means ± standard error of mean (SEM). For analysing significance of the results, a t-test based on range [39] was used. In general, p < 0.05 was regarded as significant.

## Results

### Combined *In Vivo/In Vitro* Phage Display Screening for Identification of GD2-Binding Peptides

**Fig 1:**
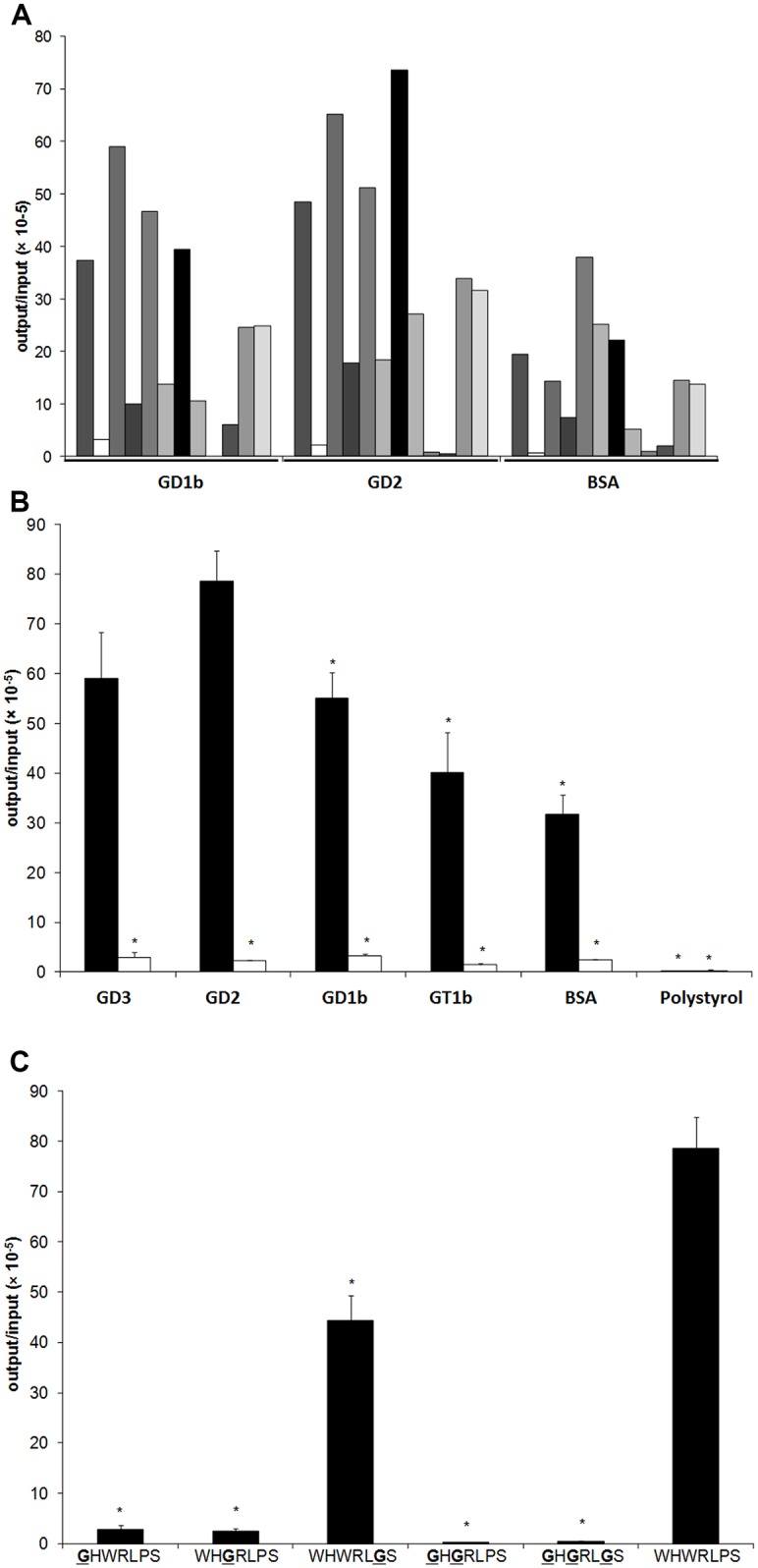
Identification of a GD2-specific binding peptide and binding characteristics of WHWRLPS-phage in vitro. **A)** Individual phage clones (n = 12) derived from a phage display screen were tested for binding to immobilized human GD1b, immobilized human GD2, or to BSA-coated surfaces. Phage clone 7 displayed the peptide sequence WHWRLPS and bound with high affinity to human GD2, and to a lesser extent, to human GD1b. **B)** Binding affinity of WHWRLPS-phage (black bars) to GD3, GD2, GD1b, GT1, BSA or polystyrol was tested and compared to control phage (white bars). “*” = p < 0.05 vs. GD2 binding of the WHWRLPS-phage. **C)** Mutations in the displayed heptapeptide and resulting affinities to GD2. * p < 0.01 vs. unmutated WHWRLPS-phage.

Specific phage ligands binding to immobilized GD2 were identified by repetitive in vitro selection. After the third round of selection, the output was used for a subsequent in vivo selection step. To this end, the phage pools were injected i.v. in mice bearing a tumour induced by s.c implantation of IMR32 cells. Phages were then recovered from the tumour tissue and were again tested for GD2 binding in vitro. One of the isolated phage clones (phage 7) displayed high affinity to GD2 and a lower affinity to GD1b, while binding to gangliosides was less specific for other clones tested (Fig 1A). Sequencing revealed that phage 7 coded for a hepta-peptide of sequence WHWRLPS.

### WHWRLPS-Phage Specifically Binds to the Disialoganglioside GD2

To characterize the specificity of the WHWRLPS-phage, we compared its binding properties to different disialogangliosides (GD3, GD2, GD1b, and GT1b) as well as unspecific binding to BSA and plastic. As an additional control for binding specificity, WHWRLPS-phage binding characteristics were also compared to a non-binding control phage containing the hepta-peptide HAIYPRH, which was designated phage 2. As expected, the control phage did not bind to any of the disialoganglioside matrices tested. In contrast, WHWRLPS-phage specifically bound to GD2 and to a lesser extent also to GD3, while binding to the other physiological gangliosides was even less specific (Fig 1B). These results indicate that the WHWRLPS-phage specifically binds to the disialoganglioside GD2.

### Glycine Mutagenesis Scanning Reveals Specificity of WHWRLPS Binding Site

In the next step, phage clones were produced in which single amino acids of the binding sequence were changed to glycine (GHWRLPS, WHGRLPS, WHWRLGS, GHGRLPS or GHGRLGS). As shown in Fig 1C, phages with an altered binding sequence showed impaired binding to GD2 compared to the WHWRLPS-phage. These results confirm that the bulky N-terminal tryptophans contribute to specific detection of the disialoganglioside GD2.

### Inhibition of WHWRLPS Phage Binding to the Disialoganglioside GD2 by Competition with the Corresponding Peptide

**Fig 2:**
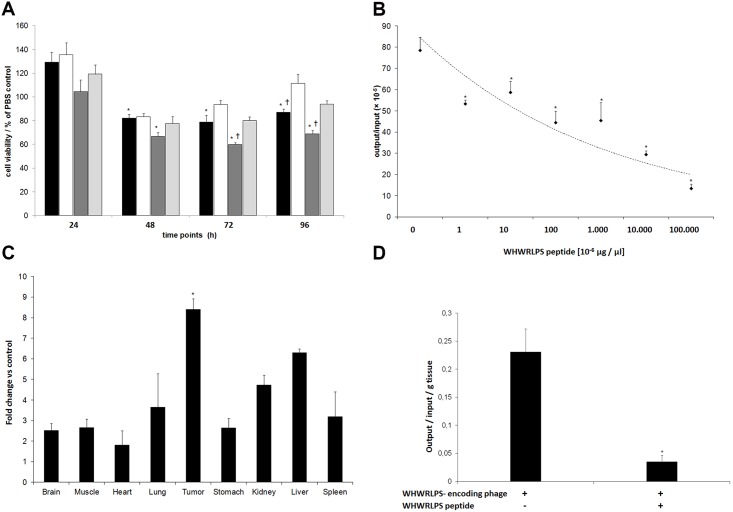
The WHWRLPS-peptide inhibits IMR32 cell viability, binds specifically to human GD2 and displays tumour homing in vivo. **A)** Cell viability of human neuroblastoma cells (IMR32) in the presence of the WHWRLPS peptide (1 μg [black] or 10 μg [dark grey]), a control peptide (1μg [white] or 10 μg [light grey]) normalised to buffer control (PBS). “*” = p<0,05 vs. PBS control, “†” = p<0,05 vs. control peptide. **B)** Competition experiments were performed in vitro to evaluated phage binding in the presence of soluble WHWRLPS-peptide. Shown are phage output titers using different peptide concentrations for competition. “*” = p < 0.05 vs. 0 μg peptide. **C)** WHWRLPS-phage or control phage (GHGRLPS) were injected i.v. mice bearing xenografts induced by s.c. injection of IMR32 cells. After 10 minutes of circulation the mice (n = 6 in each group) were sacrificed, the tumours and the organs were explanted and the number of phage present was determined. Results are shown as fold change compared to control phage (mean values ± SEM, “*” = p < 0.05 vs. control phage). **D)** WHWRLPS-phage and 200 μg WHWRLPS-peptide were coinjected i.v. in tumour-bearing mice (n = 6). Mice were sacrificed 10 minutes later, the tumours and the organs were explanted and the number of phage present was determined. Representative results of 3 independent experiments are shown (mean values ± SEM,”*” = p < 0.01 vs. control phage).

Next, binding of the WHWRLPS-phage to GD2 was analysed in the presence of the cognate peptide in a competition assay in vitro. We first analysed the impact of the WHWRLPS-peptide (1 μg or 10 μg/ml) on IMR32 cell viability. Maximum reduction of cell viability was observed after 96 h and 10 μg/ml peptide when compared to the same amount of an unrelated control peptide (Fig 2A). This suggests that binding of the GD2-specific peptide to IMR32 cells is sufficient to reduce cell viability. As shown in Fig 2B, the WHWRLPS-peptide induced dose-dependent reduction of phage binding with a maximum reduction of 82% in the presence of 10 μg peptide. Interestingly, a peptide concentration of as low as 100 pg/ml was sufficient to significantly reduce phage binding. These results demonstrate specificity of phage binding and add an additional layer of evidence for specific binding of the cognate peptide to the disialoganglioside GD2.

### Analyses of Tumour-Homing by the WHWRLPS-Phage *In Vivo*

Biodistribution of the WHWRLPS-phage or a control phage harbouring a modified binding motif (GHGRLPS) were analysed in vivo by comparing their tumour-homing ability. For this purpose, either WHWRLPS-phage or control phage were injected i.v. into mice bearing IMR32-induced xenografts. Significantly higher amounts of the WHWRLPS-phage could be recovered from tumour tissue in comparison to control phage, while phage recovery from other organs was lower than that of the control (Fig 2C). Notably, control phages did not accumulate in tumour tissue. We next performed a competition assay in vivo by i.v. coinjection of 200 μg WHWRLPS-peptide and the WHWRLPS-phage in three mice bearing in IMR32-induced xenografts. Phage recovery from tumour tissue was reduced by 84% in the presence of the competing peptide compared to injection of phage only (Fig 2D). These results corroborate to the findings obtained in vitro and furthermore confirm that homing of the peptide and phage coding for the WHWRLPS occur at the same binding sites in the tumour.

### Tumour-Homing of the ^68^Ga and ^111^In -Labelled DOTA-Peptide WHWRLPS

**Fig 3:**
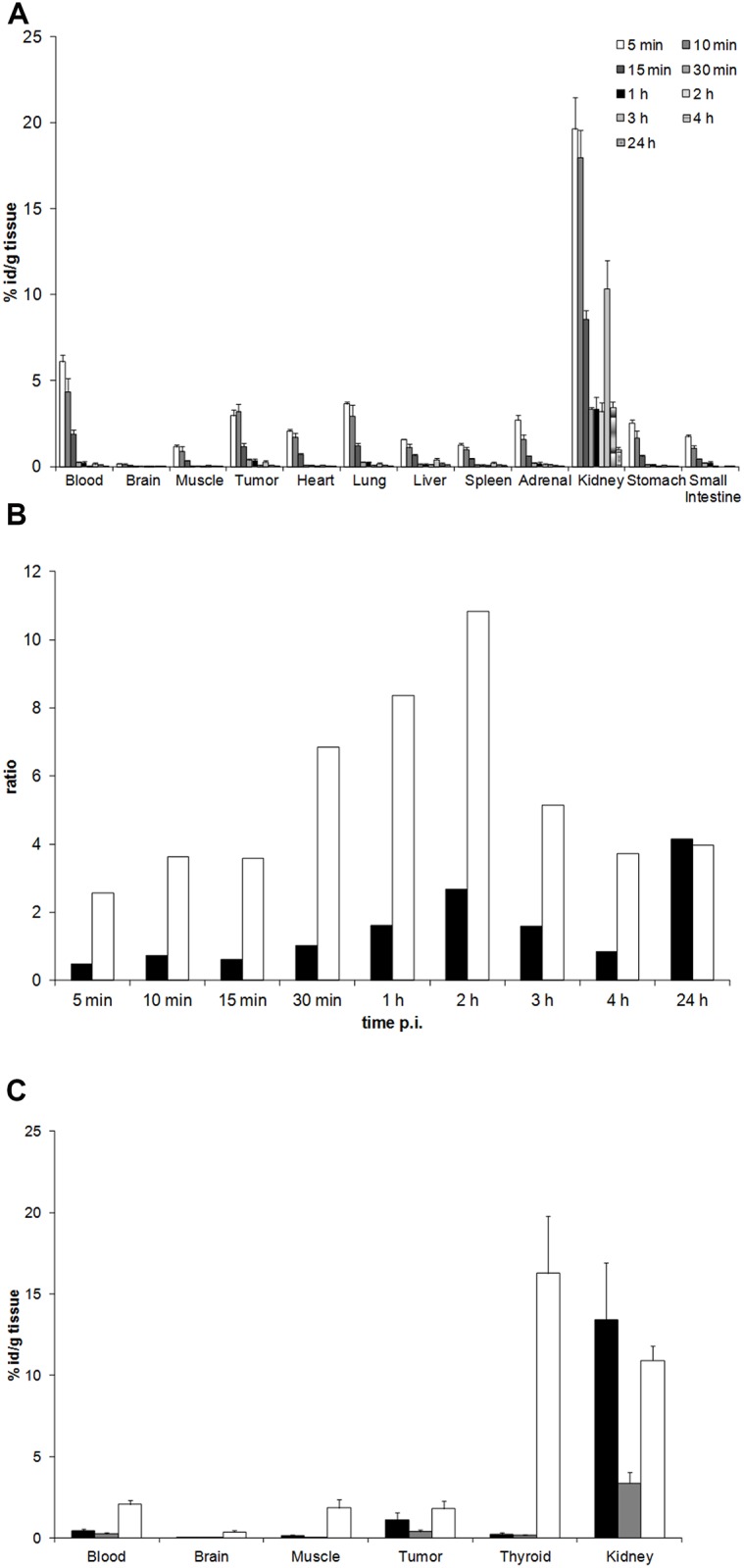
Tumour-homing of the ^68^Ga and ^111^In -labelled DOTA-peptide WHWRLPS compared to ^124^I-MIBG. **A)** The WHWRLPS-DOTA peptide was labelled with ^111^In and i.v. injected into mice (n = 3) bearing xenografts induced by s.c. injection of IMR32 cells. Mice were sacrificed at indicated time-points, tumours and organs were explanted and their activity concentration measured. Results are presented as mean values ± SEM. **B)** Tumour/blood ratio (black bars) and tumour/muscle ratio (white bars) of ^111^In- labelled WHWRLPS peptide using the data obtained in 3A, p.i. = post injection. **C)** The ^68^Ga- or ^111^In-labelled WHWRLPS-DOTA peptides (black bars and grey bars, respectively) were injected i.v. in IMR32 tumour-bearing mice (n = 3). As a control, ^124^I -MIBG (white bars) was administered to two mice. Mice were sacrificed after one hour, the tumour and the organs were explanted and their activity concentration was quantified. Results are presented as mean values ± SEM.

^111^In—labelled WHWRLPS peptides (max. 700 kBq) were injected i.v. in mice bearing IMR32-induced xenografts. Tissues and blood were collected at different time points ranging between 5 min and 24 h and their activity concentration was determined. The maximum activity concentration in tumour tissue compared to blood was observed one hour after injection (Fig 3A). As expected, unspecific accumulation of radioactivity in the kidneys was high, probably due to proximal tubular reabsorption of the peptide [40]. Calculation of the tumour/blood ratio and the tumour/muscle ratio were determined to identify the optimal time point for subsequent application in diagnostic settings (Fig 3B). One hour post injection the tumour/blood ratio of radioactivity reached a maximum value of 1.6, while the tumour/muscle ratio was 8.3 at that time point. The tumour/muscle ratio reached a maximum value of 10 two hours after injection. Comparison of the ^111^In -labelled and the ^68^Ga -labelled peptide one hour after injection revealed higher tumour-specific uptake for the ^68^Ga -labelled peptide (Fig 3C). Similarly, the effects of ^68^Ga -labelled WHWRLPS-peptide and the diagnostic standard, ^124^I -MIBG, were analysed in vivo using the same xenograft model one hour after tracer injection. As expected, accumulation was highest in the thyroid for ^124^I -MIBG and in the kidney for the ^68^Ga labelled peptide. Interestingly, the activity concentration in blood was higher for ^124^I -MIBG when compared to the ^68^Ga labelled peptide, while accumulation in tumour tissue was comparable for both tracers (Fig 3C).

### PET Imaging of Tumour Using the ^68^Ga -Labelled DOTA-Peptide WHWRLPS

**Fig 4:**
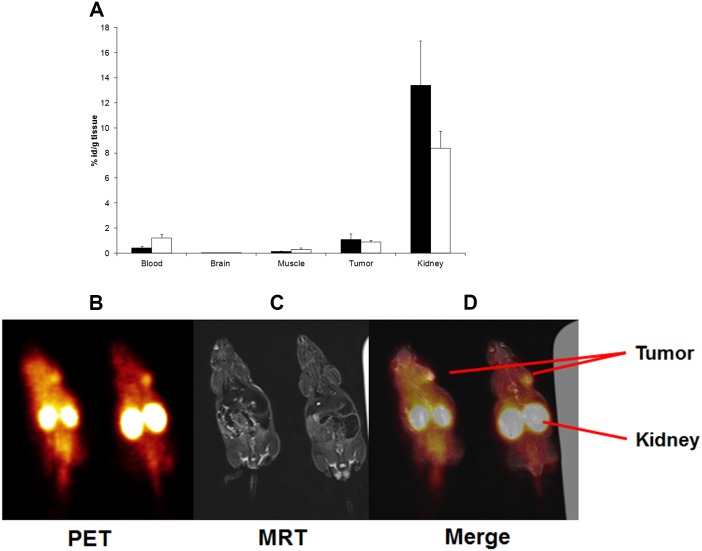
PET imaging of tumour using the ^68^Ga -labelled DOTA-peptide WHWRLPS. **A)** For kidney protection, a mixture of Gelofundine, lysine and arginine was i.v. injected in tumour-bearing mice prior to i.v. injection of the ^68^Ga-labelled WHWRLPS peptide. After one hour, mice were sacrificed, tumours and organs were explanted and their radioactive content was quantified. All values were corrected for tissue weight and radioactive decay. Results are presented as mean values ± SEM (black bars: no kidney protection, white bars: with kidney protection as described above). **B)-D)** The ^68^Ga-labelled WHWRLPS peptide was i.v. injected in IMR32 tumour-bearing mice. After one hour, peptides were detected using a PET/MRT scanner. **B)** PET image, **C)** MRI image and **D)** merge of B) and C).

Next, experiments using a diagnostic setting and PET imaging were conducted. For these studies, ^68^Ga-labelled peptides were used, which displayed higher uptake across all tissues in comparison to ^111^In–labelled peptides. We first analysed the effects of kidney protection by injection of Gelofusine, lysine and arginine prior to injection of the tracer. Levels of radioactivity were unaltered in tumours, but the radioactive load in the kidneys was reduced. On the other hand, kidney protection was accompanied by a higher accumulation of radioactivity in the blood (Fig 4A), although this did not reach statistical significance. One hour after injection of ^68^Ga-labelled peptides into mice bearing IMR32-induced xenografts, static pictures were taken (Fig B–4D). Analyses of this picture series revealed tracer accumulation in tumour tissue and also in the kidneys.

## Discussion

Expression of the disialoganglioside GD2 is observed in tumours of neuroectodermal origin, including neuroblastoma. As normal cells—with the exception of peripheral neurons- are GD2-negative, this molecule holds great promise in serving as an exceptional target for diagnosis, imaging and therapy of GD2-positive cancers. Here, we aimed to identify peptides with high specificity for GD2 and to evaluate their distribution in preclinical neuroblastoma models in vitro and in vivo. For this purpose, we deployed a combined in vivo/in vitro phage-display screening approach to ensure high target specificity and tumour homing ability using xenografts of human neuroblastoma in mice. We identified a phage motif with high affinity for GD2 but lower affinity to the physiological gangliosides GD1b and GT1b. The same peptide was found to bind to the melanoma specific ganglioside GD3 [41], albeit with intermediate affinity. The binding of the phage to GD2 could be blocked by using the cognate peptide, thus confirming specificity of phage binding to tumour-derived GD2. Modifying the phage binding motif resulted in reduced affinity for GD2, especially when the N-terminal tryptophan moieties were converted to glycine. Proliferation of neuroblastoma tumour cells in vitro was decreased in the presence of GD2-binding peptide. This is in line with findings using GD2 specific monoclonal antibodies, which have been reported by Doronin and co-workers to be also directly cytotoxic [27]. The mode of cell death was not completely elucidated, but was specifically mediated by GD2-mAb binding to GD2 and the cytotoxic effect correlated with the GD2 content of the cell [27]. Thus, in vitro results suggested that we obtained a phage and a peptide motif specific for the disialoganglioside GD2.

In vivo, phages displaying the GD2-binding peptide accumulated in xenografts of human neuroblastoma. This phage binding could be inhibited by co-injection of the cognate peptide confirming the tumour homing ability of both, the phage and the peptide. Phage accumulation was detected in the liver, the spleen and, to a lesser extent, in the lung. Most likely, this unspecific uptake of WHWRLPS-phage can be attributed to the mononuclear phagocytic system (MPS) in these organs [42]. This is corroborated by the fact that the control phages followed the same distribution pattern [43] [44]. For pharmacokinetic analyses, we used ^111^In labelled DOTA coupled WHWRLPS peptide in xenograft tumour bearing mice and found a strong short-term enrichment in the tumour as well as in the kidneys. Comparison of ^68^Ga and ^111^In DOTA-labelled peptides one hour after tracer injection revealed higher specific uptake of the ^68^Ga-DOTA labelled peptide in the tumour tissue. It remains to be elucidated, why the ^111^In-labelled peptide showed a general lower uptake in the measured tissues. Comparison of the clinical standard tracer for diagnosing neuroblastoma, ^124^I-labelled MIBG (metaiodobenzylguanidine) [45], to the ^68^Ga-labelled DOTA coupled WHWRLPS peptide revealed comparable patterns of tumour uptake. The high uptake of ^124^I-labelled MIBG in the thyroid may be caused by free iodine due to de-iodination of the tracer, since the thyroid was not blocked in this experiment. The ^68^Ga-labelled peptide showed substantially higher uptake in the kidneys compared to ^124^I-MIBG (Fig 3C), which could be reduced by injection of a mixture of Gelofusine, lysine and arginine. Reduced uptake in the kidney was accompanied by increased retention of the ^68^Ga-labelled peptide in the blood. For simulation of a diagnostic setting, ^68^Ga -labelled DOTA coupled WHWRLPS peptide was injected into mice bearing s.c. xenografts induced by IMR32 neuroblastoma cells. Using a clinical PET/MRT scanner, the uptake pattern of the ^68^Ga -labelled DOTA coupled WHWRLPS peptide was similar to the uptake pattern found in the ex-vivo measurements. As intense uptake in the kidneys was the most prominent feature of the PET images, kidney protection should be taken into consideration for optimizing the potential diagnostic use of the labelled peptide and for reducing the radiation exposure. Amino acid infusions have been recommended as nephroprotection for peptide receptor radiotherapy of neuroendocrine tumours (DOTA-TOC) [46–48]. In light of high kidney uptake despite amino acid infusion, coupling of the peptide to PEG chains to change the excretion route of the peptide should be evaluated, especially if a therapeutic use will be envisioned.

Taken together, we here describe identification, preclinical evaluation and pharmacodynamics of a peptide, which specifically binds to the human disialoganglioside GD2. Tumour-specific uptake of a ^68^Ga-labelled peptide was comparable to the clinically used ^124^I -MIBG at one hour after tracer injection. We envisage coupling of this peptide to magnetic nanoparticles or cytotoxic agents to further evaluate these in diagnostic and therapeutic settings, respectively, in preclinical models of neuroblastoma.
